# Co-occurrence of sickle cell disease and oculocutaneous albinism in a Congolese patient: a case report

**DOI:** 10.1186/s13256-021-03214-5

**Published:** 2021-12-19

**Authors:** Benoît Mbiya Mukinayi, John Mpoyi Kalenda, Didier Kalombo Kalenda, Ghislain Disashi Tumba, Béatrice Gulbis

**Affiliations:** 1 Pediatrics Department, Faculty of Medicine, University of Mbujimayi, 06201 Mbuji-Mayi, Democratic Republic of Congo; 2 Sickle Cell Reference Center, Clinique Pédiatrique de Mbujimayi, Pediatrics Clinic of Mbujimayi, 06201 Mbuji-Mayi, Democratic Republic of Congo; 3 Internal Medicine Department, Faculty of Medicine, University of Mbujimayi, 06201 Mbuji-Mayi, Democratic Republic of Congo; 4 Clinical Chemistry Department, Hereditary Red Blood Cell Disorders, LHUB-ULB, Université Libre de Bruxelles, 1070 Brussels, Belgium

**Keywords:** Sickle cell disease, Oculocutaneous albinism, Mbujimayi, Democratic Republic of the Congo

## Abstract

**Background:**

Sickle cell disease and oculocutaneous albinism are rare autosomal recessive disorders both related to mutations on chromosome 11. The diagnosis of patients suffering from both pathologies is necessary to enable dedicated monitoring of any complications at the ophthalmic and skin level. However, few cases are described in the literature.

**Case presentation:**

A 14-month-old Congolese male child affected by oculocutaneous albinism, presented with pallor and jaundice. Blood indices revealed severe hemolytic anemia, which led to the diagnosis of sickle cell disease. The patient received a blood transfusion and close follow-up.

**Conclusions:**

The co-inheritance of sickle cell disease and oculocutaneous albinism is a reality in the Democratic Republic of Congo, although it is rarely described. Given the current state of our knowledge, specific surveillance, specifically regarding cutaneous and ophthalmological complications, should be offered in this particular population. To enable this dedicated follow-up, sensitization to screening for sickle cell anemia in albino individuals should be carried out.

## Background

Sickle cell disease (SCD) and oculocutaneous albinism (OCA) are both autosomal recessive monogenic conditions with a high prevalence in populations of sub-Saharan Africa [1–3]. Sickle cell disease is an inherited disease affecting hemoglobin, and is associated with a high risk of morbidity and mortality [4]. Its origin is linked to a point mutation in the globin gene located on chromosome 11 (11p 11-5) giving rise to abnormal hemoglobin, called hemoglobin S (HbS) [5]. The disease affects 20–25 million people worldwide, among which 50–80% of those born in sub-Saharan Africa die before the age of five if they do not benefit from optimal medical monitoring, including prevention of infectious disease [6, 7]. In the Democratic Republic of Congo (DRC), as highlighted by recent epidemiological data, 2% of newborns are homozygous for hemoglobin S [8, 9]. Although this figure is epidemiologically significant, the disease remains unknown, with a high mortality rate in a country with limited resources [10]. However, while management begins with a diagnosis of the condition, screening at birth is not at all or rarely performed [9–12].

Sickle cell disease is a multisystem disease with polymorphic involvement including skin ulcer and ocular complications. The latter are related to ischemic retinopathy and neovascularization [13–15]. OCA is a group of autosomal recessive disorders of melanin biosynthesis, characterized by a generalized reduction in the pigmentation of the eyes (oculo-), skin (-cutaneous), and hair [2, 16].

There are OCA subtypes in which the mutations are in different genes on chromosome 11 [17]. Four subtypes are described, including TYR on chromosome 11q14 for OCA 1, OCA 2 (P) for OCA 2, TYRP1 for OCA 3, and SLC45A2 (MATP) for OCA 4. Syndromic forms include Hermansky–Pudlak syndrome, linked to the HPS-1 gene, and Chediak–Higashi syndrome, linked to the CHS-1 gene [17]. In sub-Saharan Africa, it is estimated that at least 1 in 4000 people have OCA [18]. In the DRC, the most important prevalence is in Kasai [18]. Although this is difficult to prove, this is probably due to a major consanguinity among the tribes living in this region [19].

The two diseases, SCD and OCA, share common signs that can sometimes be confusing in diagnosing complications, such as depigmentation of the skin, pallor in the case of anemia, and ophthalmic or skin lesions. A leg ulcer, a sickle cell complication, can progress to skin cancer in people with albinism. There is, therefore, an important place for the education of these patients. It is understood that not being exposed to the sun makes it possible to avoid deleterious dehydration for a sickle cell patient and to prevent cancerous skin lesions in an albino patient.

The comorbidity of SCD and OCA was recently described in DRC in a study conducted in Kisangani, a city in northeastern DRC. The authors conclude that it is important to raise awareness about sickle cell disease, and the significance of prenuptial screening in this population as the sickle cell trait was found [20].

This case report aims to draw physicians’ attention to the comorbidity of sickle cell disease and OCA, and the need to screen all albinos patients for sickle cell disease in view of proposing a dedicated health education and follow-up.

## Case presentation

**Fig. 1:**
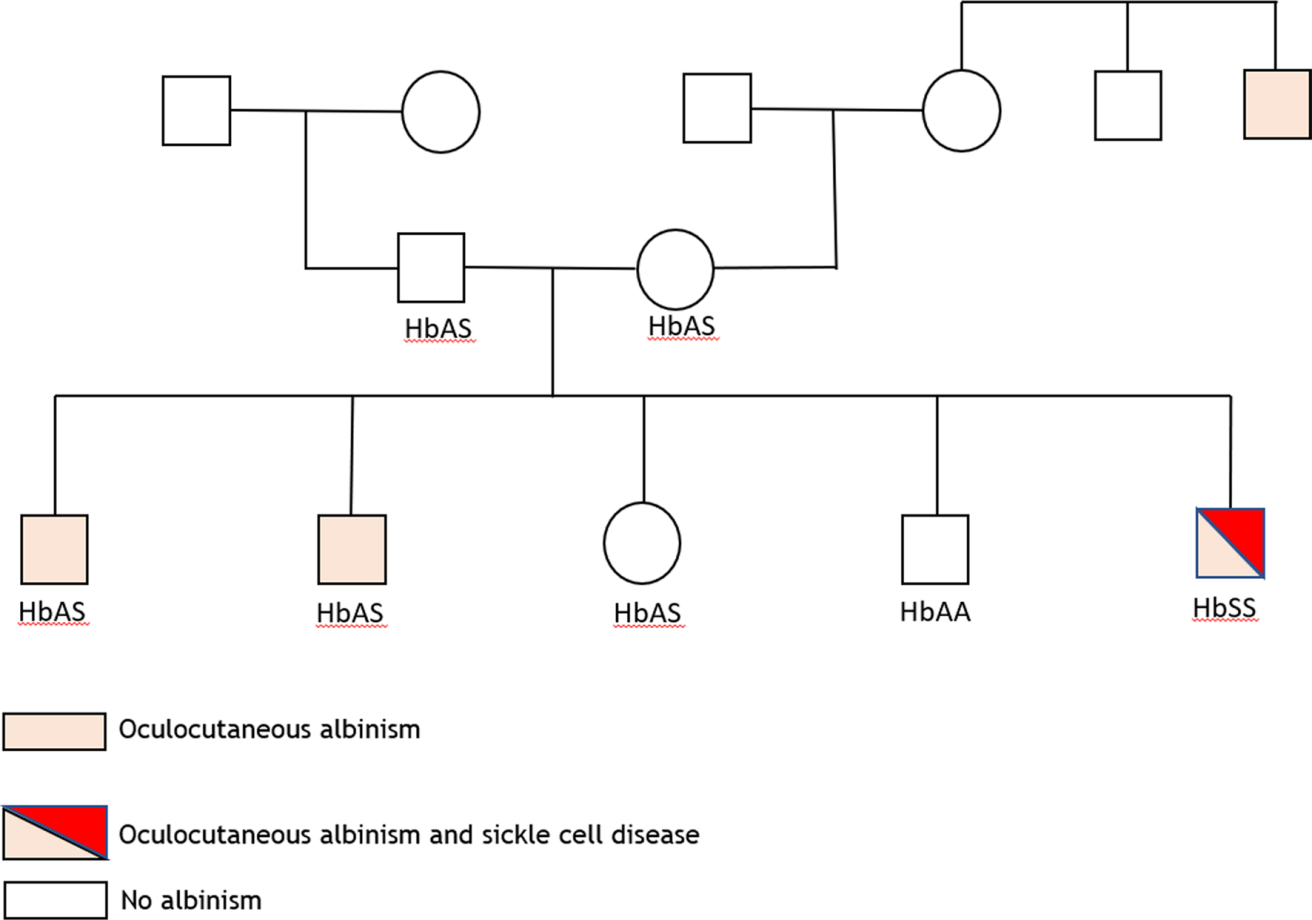
Genealogical tree

A 14-month-old Congolese male child with oculocutaneous albinism visited the Mbujimayi pediatric clinic with fever and fatigue. He was originally from Kasai Oriental, a region of the Democratic Republic of the Congo. He was the youngest in a family of five children, three of whom had oculocutaneous albinism. He himself has sickle cell disease and the rest of the siblings have a sickle cell trait (Fig. 1). He was born at term with a birth weight of 3000 g, and presented with spontaneously resolutive neonatal jaundice. The other significant history was episodes of fever with a monthly frequency of two episodes. No vaso-occlusive crisis was clearly diagnosed, in particular no dactylitis episode was reported. The vaccination schedule according to the expanded program of immunization in the DRC was respected with Bacille Calmette et Guérin (BCG) vaccine against tuberculosis; diphtheria, tetanus and pertussis (DTP) vaccine; oral polio vaccine (OPV) against polio; hepatitis B and haemophilus influenzae vaccine (HepB-HiB1); rotavirus vaccine (Rotasiil1); pneumococcal vaccine (Prevenar); measles vaccine (VAR); and yellow fever vaccine (AAV). The patient has never been hospitalized and has always been treated on an outpatient basis in health centers during febrile episodes such as malaria or typhoid fever. The siblings reported no particular clinical history.

His parents are not albinos and have no specific medical history; they never benefited from a prenuptial test. They reported their fear of the judgment of others because they have albino children when they are not affected.

**Fig. 2:**
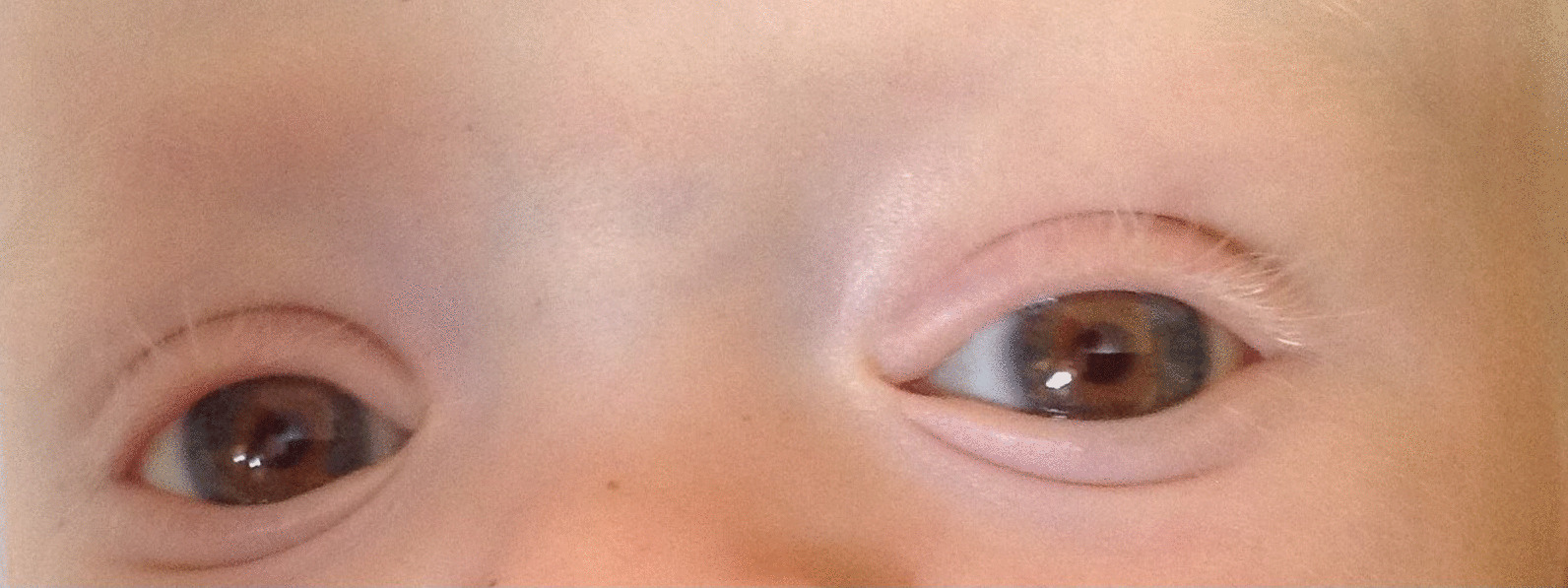
Physical examination that showed very light skin with blond hair and eyebrows, dark brown eyelashes and irises

The patient was wide awake with good contact and interaction. Generalized pallor was noted with subicteric conjunctivae, and the irises were bluish gray and translucent, thus appearing red with nystagmus (Fig. 2). The patient’s skin was pinkish white, depigmented, without any particular lesions or bruises.

On physical examination, weight and height were at the third and tenth percentile for age, respectively. There was no fever (temperature 36.5 °C), the respiratory rate was high (53 cycles/minute), as well as the heart rate (176 beats/minute), but the latter was regular with the presence of a 1/6 systolic murmur at the mitral focus. The oxygen saturation was 94%. The oral examination was normal. The lymph nodes were free, without lymphadenopathy. The lung examination was normal. The abdomen was supple and painless. There was stage III splenomegaly according to the Hackett classification. Laboratory tests showed a hemoglobin level of 48 g/L and white blood cells at 13,200/μl (complete blood count performed with the Sysmex poch-100i; Sysmex, Norderstedt, Germany).

The diagnosis of SCD has been suggested on the basis of pallor, jaundice, and severe anemia. It was confirmed first by a positive rapid test (BioMedomics, Inc, Morrisville, USA) and then by hemoglobin electrophoresis.

The diagnosis of albinism was proven by molecular genetics on a blood sample and identification of the mutation involved, that is homozygosity for the 2.7 kb deletion of OCA2 (laboratory “Centro Nacional de Biotecnologia CNB-CSIC Campus de Cantoblanco, Darwin 3, 28049 Madrid, Spain). A family investigation was then performed (see Fig. 1).

Given the very low hemoglobin level, the patient’s age, and SCD, a blood transfusion was indicated. Antibiotic treatment (ceftriaxone and amikacin) was started in the hospital. The evolution was favorable. On discharge from hospital, routine prophylaxis with folic acid and oral penicillin was initiated, and advice on  crisis prevention and medical monitoring was given to the parents.

A dedicated patient monitoring program has been set up for sickle cell anemia and albinism with hygiene advice and sun protection measures. The patient received a hat, sunglasses, and sun protection cream. Ophthalmologic follow-up has also been set up. The evolution remains dermatologically stable, no skin lesion was observed. For SCD, the patient received treatment with hydroxyurea since early 2020. It is still too early to objectify the benefit of this therapy but he had only one infectious episode, one vaso-occlusive crisis, and did not require blood transfusion for over a year.

## Discussion and conclusions

The purpose of this case report was to draw the attention of physicians to the possibility of comorbidity of SCD and OCA, and the need to screen for SCD in people with albinism. Clinical and biological data have shown that it was indeed a SCD and OCA comorbidity in a 14-month-old child from the province of Kasai Oriental in the DRC. Hemoglobin electrophoresis and the genealogical record demonstrated homozygosity for hemoglobin S, and although the diagnosis of OCA is generally clinical [21], genetic testing has confirmed homozygosity for the deletion 2.7 kb of OCA2. Comorbidity of the sickle cell trait and OCA was recently described in the DRC in 221 individuals. This study demonstrated that the frequency of the sickle cell trait was equivalent in the two groups (82 albinos including 18.3% carriers of HbS and 139 non-albinos including 18% carriers of HbS) [20]. This study did not report any case of SCD in this population but the authors concluded that it is important to sensitize this category of people to be screened for hemoglobin S [20]. SCD patients present with numerous acute and chronic complications that contribute to early mortality and, consequently, national medical costs [22].

The co-occurrence of SCD and OCA poses a problem to clinical follow-up mainly in terms of ophthalmic and cutaneous complications [12, 23, 24]. Albinism can lead to reduced vision, photosensitivity, nystagmus, strabismus, refractive errors, iris transillumination, macular hypoplasia, and abnormal decussation of optic nerve fibers are the most common aspects. OCA debilitates [24–26]. In SCD, it is sickle cell retinopathy that can lead to irreversible loss of vision if it is not correctly diagnosed and treated early [12, 23, 27]. The co-occurrence of SCD and OCA could deteriorate the patient’s vision more rapidly and requires special monitoring; annual ophthalmologic follow-up for sickle cell disease [12] should include assessment, in particular, of maculopathy [17]. Certain measures can be taken to improve the patient’s visual function and educate them on the importance of sun protection [28].

In sickle cell patients, skin ulcers are often debilitating, refractory to treatment, and recurrent [12]. These ulcers are precipitated by microvasculitis [29]. Furthermore, reduced skin pigmentation leads to photosensitivity and an increased risk of skin cancer in OCA [30, 31]. One study on albinism in the DRC described that squamous cell carcinoma was the most common lesion [18]. When skin ulcers are present, there is an increased risk of skin cancer [17, 32]. Skin examinations are therefore performed at 6–12 month intervals starting in adolescence.

Anemia is one of the consequences of SCD. In low-resource settings, it is the clinical assessment of pallor or any sign of its intolerance that leads to the diagnosis of severe anemia or its exacerbation [33]. However, in the presence of OCA, anemia can be difficult to detect due to the very pale color of the skin [32]. Despite the cost to the patient, in this situation a measurement of the hemoglobin level is indicated.

Genetically, SCD and OCA are rare autosomal recessive diseases caused by mutations on chromosome 11 [12, 17]. We report here the first case of a patient presenting simultaneously with SCD and OCA, suggesting the possibility of a presumed genetic link between these two rare diseases. An African family with four albinos associated with sickle cell anemia for one member and a sickle cell trait for three others was reported in 1956 [34]. This study demonstrated that if the hypothesis of simple recessive Mendelian inheritance of OCA is correct, the absolute link between the genes for albinism and the beta-globin gene does not occur, but a looser linkage might be a possibility [35].

Empirical observations are in favor of fewer vaso-occlusive crises in sickle cell patients with lighter skin compared with those with darker skin. Nevertheless, as described by Bakare *et al.*, comparing a group of patients with OCA and SCD with an SCD group without OCA could provide a feasible way to challenge this hypothesis. In the future, genetic and pharmacological interventions aimed at regulating melanin production may play a role in alleviating the severity of phenotypic expression of sickle cell patients [3].

The co-inheritance of SCD and OCA has rarely been described. In the absence of neonatal screening for SCD, its diagnosis is probably not made and specific follow-up, in particular for skin and ophthalmological complications, is not offered. This case also highlights a lack of clinical knowledge of this type of combination. Awareness-raising programs for screening for sickle cell anemia in this category of the population are necessary. In the present state of our knowledge, ophthalmologic and cutaneous complications should be the subject of an appropriate follow-up and anemia should not be underestimated.

## Data Availability

All data generated and/or analyzed during the current study are available from the corresponding author on reasonable request.

## Abbreviations

DRC: The Democratic Republic of the Congo
HbS: Hemoglobin S
OCA: Oculocutaneous albinism
NO: Nitric oxide
SCD: Sickle cell disease
